# New Aspects of MgH_2_ Morphological and Structural Changes during High-Energy Ball Milling

**DOI:** 10.3390/ma13204550

**Published:** 2020-10-13

**Authors:** Tomasz Czujko, Ewelina E. Oleszek, Mariusz Szot

**Affiliations:** 1 Institute of Materials Science and Engineering, Faculty of Advanced Technologies and Chemistry, Military University of Technology, Kaliskiego2, 00-908 Warsaw, Poland; 2 Department of Research and Development, Polish Security Printing Works (PWPW), R. Sanguszki 1 Street, 00-222 Warsaw, Poland; ewelina.kosciuczyk@gmail.com; 3 Department of Mechanical Devices Testing and Rocks, Central Mining Institute, Pl. Gwarków 1, 40-166 Katowice, Poland; mszot@gig.eu

**Keywords:** magnesium, hydride, ball milling, microstructure

## Abstract

Magnesium hydride, despite the decomposition temperature being incompatible with the operating temperature of a typical PEM cell, is still considered a prospective material for hydrogen storage. Hence, this paper presents new aspects of the influence of milling time on the structural changes and temperature of MgH_2_ decomposition, with particular emphasis on the changes taking place in the first few seconds of the milling process. This paper presents qualitative and quantitative changes in the powder particle morphology determined using scanning electron microscopy (SEM) and infrared particle size analysis (IPS) systems. The crystallographic structure of the powders in the initial state and after mechanical milling was characterized by X-ray diffraction. The decomposition temperature and activation energy were determined by the differential scanning calorimetry (DSC). Changes in the activation energy and decomposition temperature were observed after only 1–2 min of the milling process. Two basic stages of the milling process were distinguished that impacted the MgH_2_ decomposition temperature, i.e., mechanical activation and a nanostructuring process. The activation was associated with the initial stage of particle size reduction and an increase in the fraction of fresh chemically active powder particle surfaces. On the other hand, the nanostructuring process was related to an additional decrease in the MgH_2_ decomposition temperature.

## 1. Introduction

The dynamic development of the hydrogen economy creates a real alternative to conventional energy carriers. One of the barriers limiting the possibility of widespread implementation worldwide is the lack of a cheap, effective and safe method of hydrogen storage. Hydrides in solid form are a prospective method of storing hydrogen, and this approach has been dynamically developing in recent decades. Hydrides in solid form, which include metal hydrides [1,2,3], hydrides based on intermetallic phases [4,5] and chemical compounds with hydrogen (complex metal hydrides) [6,7], are characterized by a much higher volume capacity than that of hydrogen in compressed or liquefied form. Moreover, hydrides show no imperfections that characterize the other two systems, such as high-pressure safety considerations, high compression costs, large evaporation losses, safety and condensation costs. A high purity of the hydrogen, which is released from hydrides in solid form, appears to be very important because it enables a direct fuel cell supply [8].

One of the most popular metal hydrides for numerous research groups is magnesium hydride (MgH_2_). The growing interest in magnesium hydride and the development of new and effective methods of production are primarily due to the special properties of this material. Magnesium hydride is characterized by a relatively high hydrogen capacity of 7.6 wt.% H_2_, high availability and relatively low cost of production. The magnesium hydride decomposition reaction is a fully reversible reaction [9,10,11,12,13,14]. Unfortunately, the use of magnesium hydride has two major limitations, namely, the high desorption temperature of 325–425 °C and relatively slow hydrogen release kinetics [15].

The issue of eliminating thermodynamic barriers in the process of magnesium hydride decomposition is the subject of many scientific investigations. The most commonly used methods of hydride destabilization involve reducing the decomposition temperature, such as with mechanical milling, which leads to a nanostructure combined with a reduction in the particle size of the MgH_2_ powder and the use of composite additives [16]. These composite additives can act as catalysts and are a part of the so-called reactive composites [17,18,19,20,21]. Mechanical milling is currently the most commonly used method, leading to a reduction in the crystallite size, particle size, surface interaction and the distribution of catalyst materials on the Mg/MgH_2_ surface in Mg-based hydrogen storage materials [15,22,23].

Zaluska and Zaluski were two of the first researchers to investigate the effectiveness of mechanical milling of magnesium in the context of improving its hydrogen storage properties [24,25,26]. Mechanical milling introduces a number of changes in the comminuted material, including a reduction in the particle size from several dozen micrometers to ~500 nm [24], an increase in specific the surface area from 1.1 to 9.1 m^2^ [27], the generation of crystal lattice defects [28,29], a reduction in the size of crystallites to the nanometer range from 400 nm to 5 nm (the nanostructuring process of hydride powder) [24,30,31], an increase in grain boundary volume fraction [28] and the creation of “clean” oxide-free surfaces that facilitate hydrogen chemisorption (mechanical activation) [14,32,33]. Unfortunately, the mentioned changes occurring during milling of MgH_2_ powder are difficult to isolate because they occur at a similar stage of the process.

The most anticipated effect of the high-energy mechanical milling process is the grinding of powder particles. However, this is a multistage process, which is why we obtain increasingly complex changes at the individual stages. In the initial stage, some of the powder particles are heavily deformed, while the rest remain intact. The particle size distribution at this stage is quite wide. In the case of magnesium hydride, the fragmentation of particles is quite fast, while at the same time, a sharp decrease in the value of the crystallites is observed. The next stage of grinding is the alloying process, during which the agglomerates of individual powder particles are formed, thereby increasing their size. The MgH_2_ nanocrystalline particles produced in the initial phase undergo a significant increase in size at this stage, from the initial 9 nm to more than 100 nm on average [34]. In the final stage, the intense fragmentation of particles takes place, initially on the balls and the walls of the cylinder and then in the entire volume. The effect of this milling step is to obtain an even particle size distribution [35,36]. The crystallite size remains constant after the optimal milling time. It is interesting that, despite obtaining a nanostructured MgH_2_ powder after mechanical milling, there is no significant improvement in the thermodynamics of the hydrogen desorption process [37]. It is true that there are slight changes in the decomposition temperature along with the particle size reduction below 1 μm [15]. Nevertheless, Mooij and Bernard Damet [38] observed a clear destabilization of magnesium hydride when the thicknesses of Mg layer reached 3 nm. However, for MgH_2_ with micrometric particle size, it can be concluded that the improvement in the kinetics is an effect associated with a reduction in the particle size and creation of new, active surfaces on the magnesium particles, which in turn leads to an improvement in hydrogen chemisorption and a shortening of hydrogen diffusion paths [28]. Basically, the improvement in hydrogenation properties obtained in the milling process is attributed to the nanostructuring process. However, Varin et al. also mentioned the role of the powder particle size [15], noting a decrease in the MgH_2_ decomposition temperature together with a reduction in the powder particle size. However, the observed changes occurred mainly in the submicron size range and were also associated with the participation of the metastable γ-MgH_2_ phase. Moreover, this effect was observed immediately after milling. However, after the first desorption/absorption cycle, the decomposition temperature increased by approximately 20 °C regardless of the particle size, while the crystallite size increased from a dozen to several dozen nanometers.

It should be noted that the studies presented so far used similar high-energy mills and concerned milling at long times from 1–25 h [23,34,36,39,40,41]. In addition, the observed kinetics due to changes in both the particle size of the powder particles and crystallites were similar. For this reason, it is difficult to clearly determine which of the structural parameters, namely the grain or particle sizes, play a decisive role in improving the sorption kinetics and reducing the temperature of hydride decomposition. Unfortunately, the first powder samples were usually tested after a milling time of not less than 10 min. Hence, we suppose that significant changes accompanying the process of high-energy milling occurred in the first few dozen seconds.

Therefore, it seems necessary to organize the knowledge regarding the role of grain and particle sizes and the dynamics of changes in these parameters during the process of mechanical milling. In this work, we present the results of structural transformations that take place in commercially available MgH_2_ after mechanical milling over a wide range of times from 10 s to 5 h. Changes in the crystallite and powder particle sizes, decomposition temperature and activation energy as a function of milling time were studied. In addition, the influence of the structural parameters of milled MgH_2_, i.e., the impact of the particle and crystallite sizes on the decomposition temperature and activation energy, was studied.

## 2. Materials and Methods

Magnesium hydride powder (−325 mesh, 98% purity) purchased from Alfa Aesar (Ward Hill, MA, USA) was ball milled in a Fritsch Pulverisette 7 planetary mill (Fritsch, Idar-Oberstein, Germany) for 10 s, 30 s, 1 min, 2.5 min, 5 min, 15 min, 45 min, 75 min, 135 min and 300 min. The as-received powder was loaded with ten stainless-steel balls that had a diameter of 10 mm into a 20 mL bowl made of hardened steel. The ball-to-powder weight ratio was 20:1, and the rotational speed of the milling bowl was 650 rpm. For milling times longer than 15 min, a single milling cycle was 15 min, and the interval between individual cycles was 30 min. The powder, before and after milling, was kept in a Labmaster Workstation (MBraun Inert-Gas Systeme GmbH, Garching, Germany) under a continuously purified argon atmosphere with < 0.1 ppm O_2_ and H_2_O vapor.

Morphological examination of the as-received and milled powders was conducted with high-resolution SEM in a Quanta 3D FEG Dual Beam instrument (Quanta, Hillsboro, OR, USA) using secondary electrons SE and back-scattered electrons BSE detectors.

The granulometric analysis of the powders was performed using an infrared particle size (IPS) analyzer (KμK, Warsaw, Poland), which enabled the quantitative determination of the mean particle size and distribution. Moreover, the specific surface area of the powders was determined by the Brunauer–Emmett–Teller (BET) method using the nitrogen adsorption method at the temperature of liquid nitrogen on a Gemini 2360 (Micromeritics GmbH, Unterschleissheim, Germany) device.

The crystallographic structure of the powders in the initial state and after mechanical milling was characterized by X-ray diffraction (XRD) on a Rigaku ULTIMA IV (Rigaku Corporation, Tokyo, Japan) using CoKα1 radiation generated at an accelerating voltage of 40 kV and a current of 30 mA. The scan range was from 2θ = 20°–120°, and the scan rate was 1.2°/min. The X-ray profiles were analyzed with PDXL Ver.2.0 software (Rigaku, Tokyo, Japan) that incorporated the PDF-4 database. The crystallite size of the as-received and milled powders was calculated on the basis of the width of the diffraction peaks using the Hall formula.

The thermal behavior of the powders was studied by differential scanning calorimetry (DSC) (Sensys EVO -SETARAM, SETARAM Instrumentation) using ~ 20mg samples with heating rates of 2, 5, 10 and 15 °C/min and a helium flow rate of 10 mL/min. The activation energy of the MgH_2_ decomposition process was determined on the basis of DSC curves using the Kissinger equation:

(1) $$ln\left(\frac{\beta }{T_{m}^{2}}\right)=\;\frac{-E_{a}}{RT_{m}}+const$$

where *E_a_* is the energy of activation, *β* is the heating rate (in K min^−1^) of the DSC experiment, *T_m_* is the temperature at the differential heat flow maximum of the DSC experiment, and *R* is the universal gas constant (8.134472 J/(mol*K)).

## 3. Results and Discussion

Depending on the type of ball mill (the energy input during the milling process), the typical milling time for magnesium or magnesium hydride ranges from 15 min to 20 h for high-energy mills [9,11,24,25,30,42] and from 20 to 150 h for low-energy mills. [1,15]. Regardless of the type of mill used, most researchers have focused on the final morphological, structural and thermodynamic effects typical for long milling times. In this work, we also considered the changes take place in the first few seconds, which sheds new light on the dynamics of changes taking place in the material and their influence on its thermodynamic properties. The thermal stability of magnesium hydride, expressed by the decomposition temperature, was related not only to the technological parameter, which is the milling time, but also to structural changes expressed by changing the size of the powder particles and the size of the crystallites. The analysis also considered the changes in activation energy.

### 3.1. Morphological and Structural Changes during MgH_2_ Ball Milling

**Figure 1:**
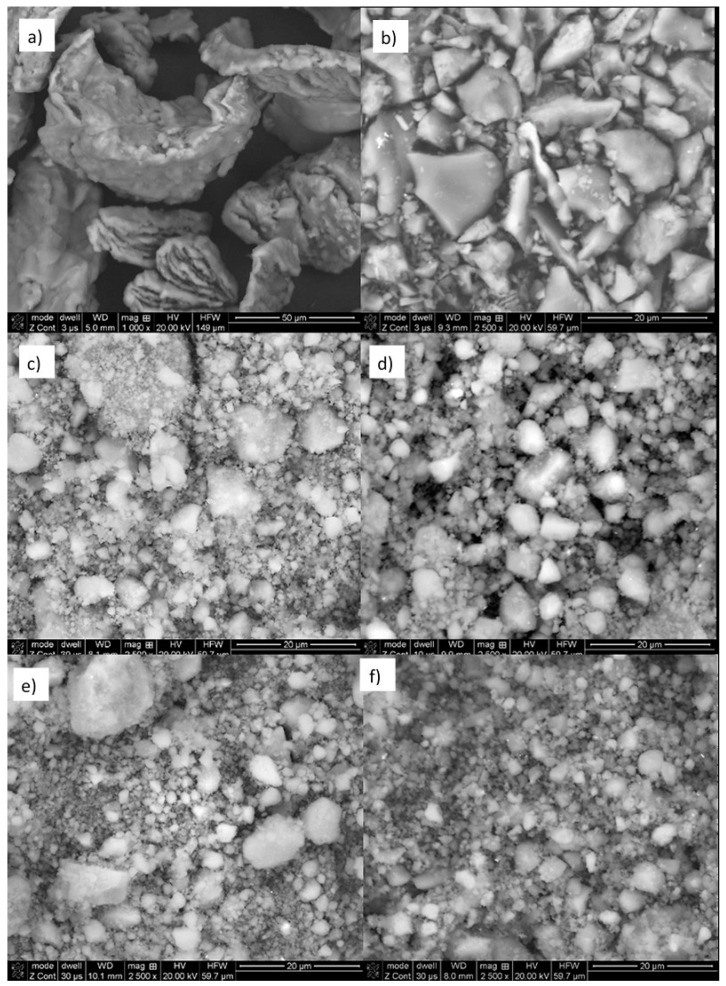
The morphology of the MgH_2_ powders: as-received (**a**) and after milling for 10 s (**b**), 15 min (**c**), 45 min (**d**), 135 min (**e**) and 300 min (**f**).

The morphology of the as-received and milled magnesium hydride powders, presented in Figure 1, was characterized by the presence of flakelike and irregular globular particles, respectively. It should be noted that a clear reduction in the particle size of the powder and a change from flakelike to globular shapes were observed after only 10 s of milling. Additional morphological changes were essentially reduced to a nonsignificant reduction in the average particle diameter and an increase in the amount of finely crushed particles. Changes in the morphological parameters of the powders are presented in Table 1.

Mechanical milling of magnesium hydride resulted in strong particle refinement. After just 10 s of milling, the average particle size was reduced from D_n_ ≈ 30 µm for the as-received powder to a level of approx. 10 µm after high-energy milling. The obtained mean particle size values indicate that this material remained at a similar level of refinement in the size range from 8.9–10.1 µm throughout the milling process that lasted up to 5 h.

The specific surface area of the magnesium hydride, determined by the BET technique, was approximately 1 m^2^/g. As in the case of IPS, the BET analysis showed a clear refinement of the powder particles even with a short milling time. Already after 10 s of milling, a clear increase in the development of the specific surface was observed. Extending the milling time even up to 300 min did not cause significant changes in the powder particle size, although the best morphological parameters (the smallest diameter D_n_ and the largest specific surface area) were obtained for powders milled for 15 min. This may be due to the tendency to form small particle conglomerates as the grinding time increased.

**Figure 2:**
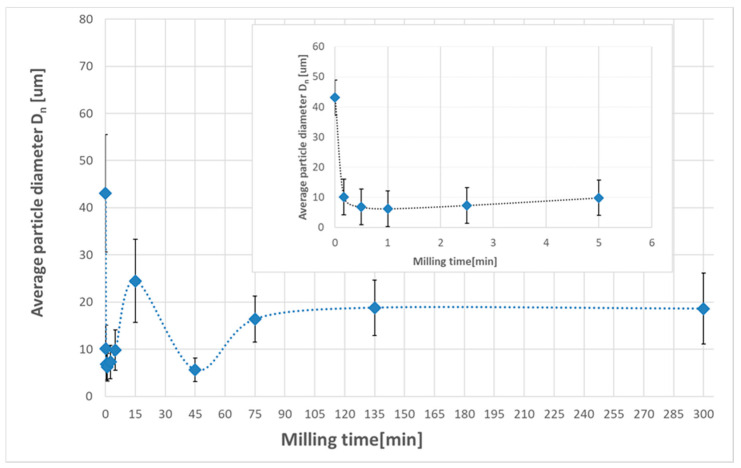
The changes in the average particle diameter D_n_ for as-received and milled MgH_2_ powders.

Similar morphological changes were observed by many other authors [1,18,22], although, for the first time, we demonstrated that they occurred in a dynamic way and were visible after only a few seconds of milling (Figure 2). Significant particle size changes generally occurred during the first 1–2 min of the milling process. On the other hand, additional milling resulted in an increase in the proportion of finely crushed particles, a reduction in the diameter of the remaining coarse particles and homogenization and spheroidization of the powder morphology.

**Figure 3:**
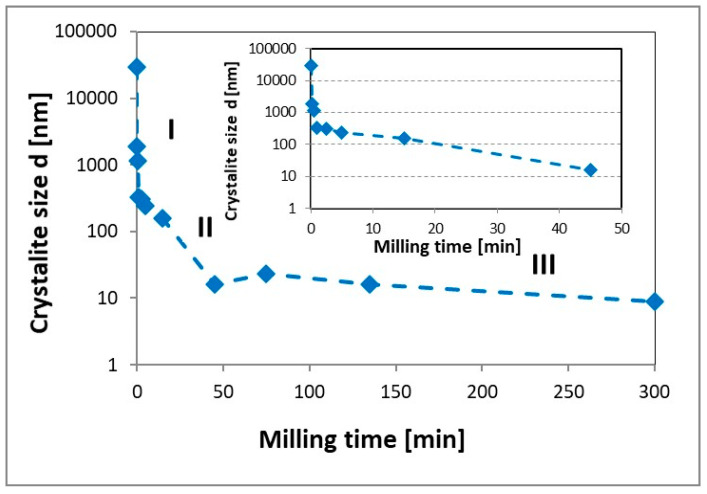
The changes in the crystallite size for as-received and milled MgH_2_.

The nanostructuring process was indicated by a reduction in the crystallite size (Figure 3) and had three stages. In the first stage (I), lasting 1–2 min, we observed a rapid reduction in the crystallite size from approx. 30 mm to 300 nm, which may be related to a dynamic reduction of the powder particle size (Figure 2). During the second stage (II), which is typical and described many times in the literature [24,43], a nanostructuring process from 10–20 nm lasted approx. 45 min for the used milling parameters. Additional milling of the MgH_2_ powder (stage III) did not significantly reduce the crystallite size.

Because, during the cycles, magnesium hydride does not have a tendency to decrepitate and forms conglomerates of small particles [1], the crystalline size change that took place in the first stage was related to the powder particle size and is permanent and independent of the number of cycles or the operating temperature. However, the changes that took place in the second stage were related to the formation of nanostructures within individual powder particles and determined by the thermal stability of the material [1].

### 3.2. Thermal Decomposition of the MgH_2_ after Ball Milling

To determine the effect of mechanical milling and the related structural changes on the temperature and kinetics of the MgH_2_ decomposition process, which are determined by the activation energy and the decomposition temperature (T_on_ and T_peak_), calorimetric tests were carried out. The T_on_ value indicates the temperature for the beginning of the hydride decomposition, while T_peak_ is the temperature value at which the decomposition rate reaches its maximum rate.

**Figure 4:**
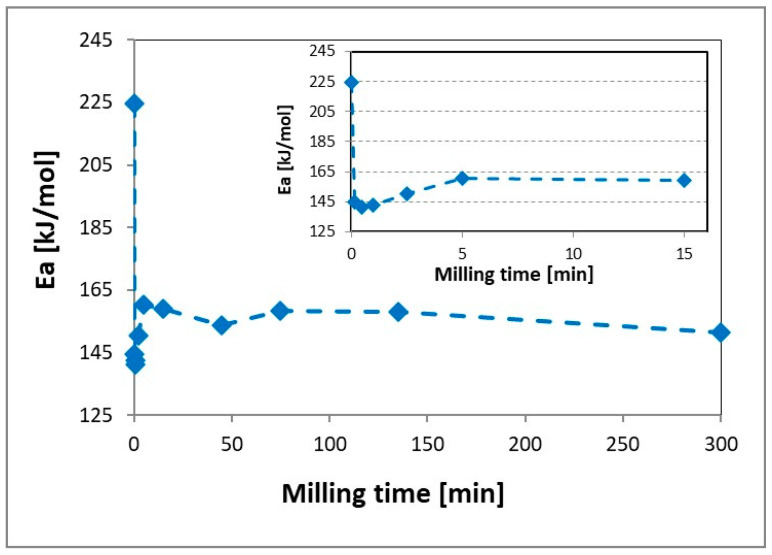
The changes in the activation energy E_a_ for MgH_2_ in as-received and milled forms.

The changes in the activation energy of the magnesium hydride decomposition process presented in Figure 4 also had two stages. The activation energy determined for magnesium hydride in the as-received form is comparable with that in the literature [1,44] and reflects the degree of contamination of the powder particle surface with impurities in the form of magnesium oxide and hydroxide.

The sharp drop in the activation energy accompanied the initial first stage of the milling process also presented in Figure 3 and is associated with the formation of oxide-free fresh surfaces resulting from the cracking of powder particles. Further milling to produce a nanostructured powder caused a slight increase in the activation energy that remained constant even up to 300 min of milling. Similar changes in the activation energy were observed by Varin et al. [45], where a decrease in the activation energy value was nonmonotonic and a significant increase in the milling time resulted in an increase in the activation energy. However, in previously published works, these changes were not observed in a milling time as short as 1 min. It should also be noted that the observed nonmonotonic activation energy changes occurred for the milling times when the material was still in submicrometric form or at the beginning of nanostructuring process (5 min).

**Figure 5:**
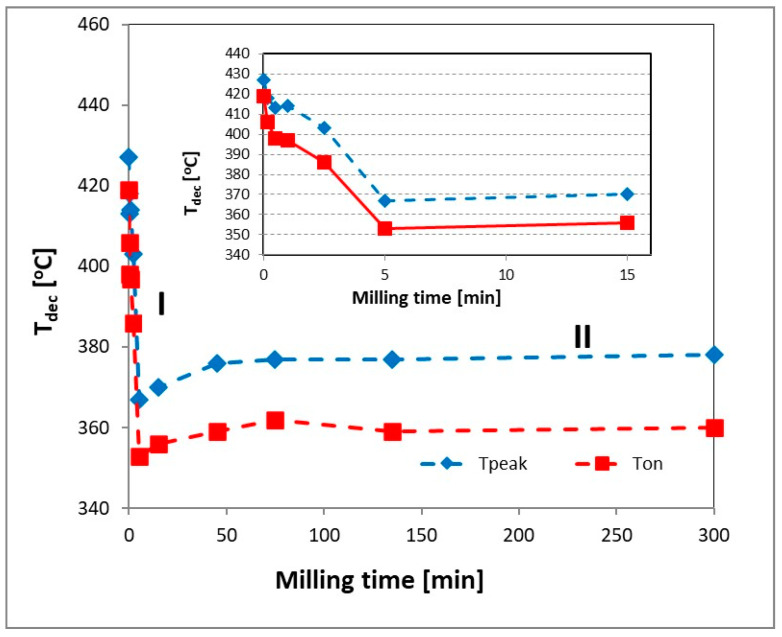
The changes in decomposition temperature for as-received and milled MgH_2_.

Changes in the decomposition temperature of magnesium hydride as a function of milling time are shown in Figure 5. As in the case of the crystallite size, three stages were observed.

In the first stage of milling (0–5 min), we observed a sharp decrease in the MgH_2_ decomposition temperature from 427 °C to 413 °C and 367 °C to 353 °C for T_peak_ and T_on_, respectively. Additional milling caused a stable but slight increase in the decomposition temperature. Contrary to previously published results [1,9,11,15,18,31], the observed changes in the decomposition temperature occurred after only 5 min of milling and did not change with increasing milling time. This means that, from a technological point of view, the use of long milling times does not have a significant effect on a reduction in the decomposition temperature of magnesium hydride and is not justified. Moreover, a careful analysis of the changes for short grinding times (insert in Figure 5) shows that the first noticeable decrease in the decomposition temperature of magnesium hydride was already observed for 30 s of milling. This decrease clearly correlates with the decrease in the activation energy (Figure 4), which may indicate that the initial reduction in the decomposition temperature is related to a decrease in the activation energy value. On the other hand, an additional decrease in the value of the decomposition temperature with an increase in the grinding time was related to the nanostructuring process.

**Figure 6:**
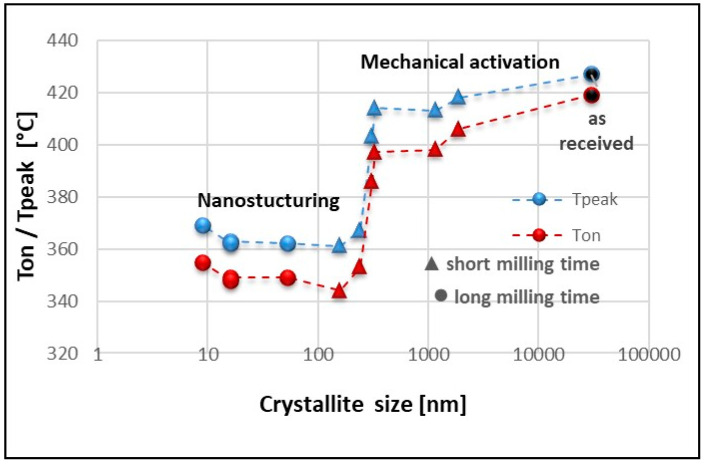
The changes in decomposition temperature for MgH_2_ vs. crystallite size.

The observed dependence suggests the need to analyze the decomposition temperature of magnesium hydride as a function of the crystallite size, as presented in Figure 6. For the material in the polycrystalline and submicrometric form (up to 300 nm), we observed a slight but noticeable decrease in the decomposition temperature related to a decrease in the activation energy. This step is typical for mechanical activation and, as already mentioned, is associated with an increase in the fraction of fresh surfaces of chemically active powder particles. With a decrease in the size of the crystallites to a nanometric size (100 nm), we observed an almost linear relationship between the size of the crystallites and the decomposition temperature, which is often presented in numerous studies regarding the influence of nanostructuring processes on the sorption properties of MgH_2_ [1,9,15,24,42].

No significant changes in the decomposition temperature were observed for powders with crystallite sizes in the range from 100–10 nm. Due to the applied milling parameters (a relatively short time of up to 5 h), the volume fraction of the γ-MgH_2_ gamma phase in the tested powders was negligible (the XRD results are not presented in this study). Hence, the presented results related essentially to the β-MgH_2_ phase. Usually, as the nanocrystalline size decreases and the milling time increases, the decomposition temperature decreases [1,9,15,24,42]. Nevertheless, these observations are typical for the first decomposition only, and in our opinion, they are related to the presence of the γ-MgH_2_ phase that reduces the stability of magnesium hydride [31]. Considering that the γ-MgH_2_ phase disappeared after the first desorption/absorption cycle, a permanent decrease in the decomposition temperature was observed for submicrometric powders with a size of approximately 100 nm.

Taking into account the changes in the size of particles and crystallites and the accompanying reduction in the MgH_2_ decomposition temperature and activation energy, we can distinguish two stages in the mechanical milling process. The first stage is related to mechanical hydride activation, which consists in a rapid reduction of the particle size and the formation of fresh chemically active surfaces. This is accompanied by a noticeable decrease in activation energy. In this work we showed for the first time that this phenomenon occurs after several seconds of mechanical milling. Usually, mechanical milling was carried out for a dozen, several dozen or even several hundred minutes and therefore, apart from mechanical activation, hydride nanostructuring was also observed [1,15,31,43]. Moreover, we have shown that with nanostructuring, there is a further reduction in the decomposition temperature of magnesium hydride. For a long milling time of 20 h, the reduction of the crystallite size is accompanied by the formation of the metastable γ-MgH_2_ phase [31]. Due to the milling applied conditions (the energy of the mill and the short milling time), the fraction of the gamma phase in our research was small and the changes in the decomposition temperature can be mainly attributed to the nanostructuring process.

## 4. Conclusions

This study investigated the effect of the milling time on changes in the structure, activation energy and decomposition temperature of magnesium hydride, with particular emphasis on the changes taking place during extremely short milling times. It was observed that significant changes occurred after 30–60 s of milling and were associated with a decrease in the activation energy caused by the formation of contamination-free powder particle surfaces. An additional decrease in the decomposition temperature was caused by the nanostructuring process. Due to the permanent reduction of the decomposition temperature, extending the milling process beyond a few minutes does not have technological justification.

## Figures and Tables

**Table 1:** Morphological properties of the MgH_2_ powder before and after milling for various times.

Properties	Milling Time						
	0 s	10 s	15 min	45 min	75 min	135 min	300 min
Diameter ^**1**^ [µm]	28.0 ± 12.8	10.1 ± 9.6	9.5 ± 8.8	9.2 ± 2.5	9.4 ± 4.9	8.9 ± 5.9	9.3 ± 5.8
SSA ^2^ [m^2^/g]	0.94	9.1	12.41	11.64	10.30	10.03	10.00

^1^ Average D_n_ diameter measured by infrared particle size analysis (IPS). ^2^ Specific surface area (SSA) measured using the BET technique.

## References

[1] Varin R.A., Czujko T., Wronski Z.S. (2009). Nanomaterials for Solid State Hydrogen Storage.

[2] Wiswall R.H., Reilly J.J. (1972). Inverse Hydrogen Isotope Effects in Some Metal Hydride Systems. Inorg. Chem..

[3] Zuttel A. (2003). Materials for hydrogen storage. Mater. Today.

[4] Kondo M., Asano K., Iijima Y. (2005). Effect of nickel addition and microstructure on absorption and desorption behavior of hydrogen in LaNi_5_. J. Alloy. Compd..

[5] Varin R.A., Zaranski Z., Czujko T., Polanski M., Wronski Z.S. (2011). The composites of magnesium hydride and iron-titanium intermetallic. Int. J. Hydrog. Energy.

[6] Jain I.P., Jain P., Jain A. (2010). Novel hydrogen storage materials: A review of lightweight complex hydrides. J. Alloy. Compd..

[7] Varin R.A., Kościuczyk E., Czujko T. (2015). Mechanical and thermal dehydrogenation of mechano-chemically synthesized calcium alanate (Ca(AlH_4_)_2_) and lithium chloride (LiCl) composite. Materials.

[8] Wronski Z., Varin R.A., Chiu C., Czujko T., Calka A. (2007). Mechanochemical synthesis of nanostructured chemical hydrides in hydrogen alloying mills. J. Alloy. Compd..

[9] Selvam P., Viswanathan B., Swamy C.S., Srinivasan V. (1986). Magnesium & magnesium alloy hydride. Int. J. Hydrog. Energy..

[10] Klyamkin S.N. (2007). Metal hydride compositions on the basis of magnesium as materials for hydrogen accumulation. Russ. J. Gen. Chem..

[11] Mushnikov N.V., Ermakov A.E., Uimin M.A., Gaviko V.S., Terent’ev P.B., Skripov A.V., Tankeev A.P., Soloninin A.V., Buzlukov A.L. (2006). Kinetics of interaction of Mg-based mechanically activated alloys with hydrogen. Phys. Met. Metall..

[12] Dornheim M., Doppiu S., Barkhordarian G., Boesenberg U., Klassen T., Gutfleisch O., Bormann R. (2007). Hydrogen storage in magnesium-based hydrides and hydride composites. Scr. Mater..

[13] Yao X.D., Lu G.Q. (2008). Magnesium-based materials for hydrogen storage: Recent advances and future perspectives. Chin. Sci. Bull..

[14] Sakintuna B., Lamari-Darkrim F., Hirscher M. (2007). Metal hydride materials for solid hydrogen storage: A review. Int. J. Hydrog. Energy..

[15] Varin R.A., Czujko T., Chiu C., Wronski Z. (2006). Particle size effects on the desorption properties of nanostructured magnesium dihydride (MgH_2_) synthesized by controlled reactive mechanical milling (CRMM). J. Alloy. Compd..

[16] Sadhasivam T., Kim H.-T., Jung S., Roh S.-H., Park J.-H., Jung H.-Y. (2017). Dimensional effects of nanostructured Mg/MgH_2_ for hydrogen storage applications: A review. Renew. Sust. Energy Rev..

[17] House S.D., Vajo J.J., Zaluzec N.J., Rockett A.A., Robertson I.M. (2017). Impact of initial catalyst form on the 3D structure and performance of ball-milled Ni-catalyzed MgH_2_ for hydrogen storage. Int. J. Hydrog. Energy.

[18] House S.D., Vajo J.J., Ren C., Rockett A.A., Robertson I.M. (2015). Effect of ball-milling duration and dehydrogenation on the morphology, microstructure and catalyst dispersion in Ni-catalyzed MgH_2_ hydrogen storage materials. Acta Mater..

[19] Czujko T., Varin R.A., Wronski Z., Zaranski Z., Durejko T. (2007). Synthesis and hydrogen desorption properties of nanocomposite magnesium hydride with sodium borohydride (MgH_2_+ NaBH_4_). J. Alloy. Compd..

[20] Pistidda C., Barkhordarian G., Rzeszutek A., Garroni S., Bonatto Minella C., Baro M.D., Nolis P., Bormann R., Klassen T., Dornheim M. (2011). Activation of the reactive hydride composite 2NaBH_4_+ MgH_2_. Scr. Mater..

[21] Zander D., Lyubenova L., Koster U., Dornheim M., Aguey-Zinsou K.-F., Klassen T. (2006). The catalytic effect of Nb_2_O_5_ on the electrochemical hydrogenation of nanocrystalline magnesium. J. Alloy. Compd..

[22] Polanski M., Bystrzycki J., Plocinski T. (2008). The effect of milling conditions on microstructure and hydrogen absorption/desorption properties of magnesium hydride (MgH_2_) without and with Cr_2_O_3_ nanoparticles. Int. J. Hydrog. Energy.

[23] Sadhasivam T., Hudson M.S.L., Pandey S.K., Bhatnagar A., Singh M.K., Gurunathan K., Srivastava O.N. (2013). Effects of nano size mischmetal and its oxide on improving the hydrogen sorption behaviour of MgH_2_. Int. J. Hydrog. Energy..

[24] Zaluska A., Zaluski L., Ström–Olsen J.O. (1999). Nanocrystalline magnesium for hydrogen storage. J. Alloy. Compd..

[25] Zaluska A., Zaluski L., Ström–Olsen J.O. (1999). Synergy of hydrogen sorption in ball-milled hydrides of Mg and Mg_2_Ni. J. Alloy. Compd..

[26] Zaluski L., Zaluska A., Ström-Olsen J.O. (1995). Hydrogen absorption in nanocrystalline Mg_2_Ni formed by mechanical alloying. J. Alloy. Compd..

[27] Aguey-Zinsou K.-F., Fernandez J.R.A., Klassen T., Bormann R. (2007). Effect of Nb_2_O_5_ on MgH_2_ properties during mechanical milling. Int. J. Hydrog. Energy..

[28] Sun Y., Shen C., Lai Q., Liu W., Wang D.-W., Aguey-Zinsou K.-F. (2018). Tailoring magnesium-based materials for hydrogen storage through synthesis: Current state of the art. Energy Storage Mater..

[29] Varin R.A., Czujko T. (2002). The effect of atomic volume on the hydrogen storage capacity of hexagonal metals/intermetallics. Scr. Mater..

[30] Huot J. (2013). Mechanochemical synthesis of hydrogen storage materials. Prog. Mater. Sci..

[31] Huot J., Liang G., Boily S., Van Neste A., Schulz R. (1999). Structural study and hydrogen sorption kinetics of ball-milled magnesium hydride. J. Alloy. Compd..

[32] Shao H., Xin G., Zheng J., Li X., Akiba E. (2012). Nanotechnology for hydrogen storage. Nano Energy.

[33] Shao H., Liu T., Wang Y., Xu H., Li X. (2008). Preparation of Mg-based hydrogen storage materials from metal nanoparticles. J. Alloy. Compd..

[34] Paik B., Walton A., Mann V., Book D., Jones I.P., Harris I.R. (2010). Microstructure of ball milled MgH_2_ powders upon hydrogen cycling: An electron microscopy stud. Int. J. Hydrog. Energy..

[35] Friedrichs O., Aguey-Zinsou K.-F., Fernandez J.R.A., Sanchez-Lopez J.C., Justo A., Klassen T., Bormann R., Fernandez A. (2006). MgH_2_ with Nb_2_O_5_ as additive, for hydrogen storage: Chemical, structural and kinetic behavior with heating. Acta Mater..

[36] Montone A., Aurora A., Gattia D.M., Antisari M.V. (2012). Microstructural and kinetic evolution of Fe doped MgH_2_ during H_2_ cycling. Catalysts.

[37] Berube V., Radtke G., Dresselhaus M., Chen G. (2007). Size effects on the hydrogen storage properties of nanostructured metal hydrides: A review. Int. J. Energy Res..

[38] Mooij L., Dam B. (2013). Hysteresis and the role of nucleation and growth in the hydrogenation of Mg nanolayers. Phys. Chem. Chem. Phy..

[39] Hanada N., Ichikawa T., Hino S., Fujii H. (2006). Remarkable improvement of hydrogen sorption kinetics in magnesium catalyzed with Nb_2_O_5_. J. Alloy. Compd..

[40] Lu H.-B., Poh C.-K., Zhang L.C., Guo Z.P., Yu X.B., Liu H.K. (2009). Dehydrogenation characteristics of Ti-and Ni/Ti-catalyzed Mg hydrides. J. Alloy. Compd..

[41] Pighin S.A., Capurso G., Russo S.L., Peretti H.A. (2012). Hydrogen sorption kinetics of magnesium hydride enhanced by the addition of Zr8Ni21 alloy. J. Alloy. Compd..

[42] Barkhordarian G., Klassen T., Bormann R. (2004). Effect of Nb_2_O_5_ content on hydrogen reaction kinetics of Mg. J. Alloy. Compd..

[43] Shixue Z., Zhang Q., Chen H., Zang X., Zhou X., Wang R., Jiang X., Yang B., Jiang R. (2015). Crystalline structure, energy calculation and dehydriding thermodynamics of magnesium hydride from reactive milling. Int. J. Energy Res..

[44] Han J.S., Pezat M., Lee J.Y. (1987). A study of the decomposition of magnesium hydride by thermal analysis. J. Less Common Met..

[45] Varin R.A., Jang M., Czujko T., Wronski Z.S. (2010). The effect of ball milling under hydrogen and argon on the desorption properties of MgH_2_ covered with a layer of Mg(OH)_2_. J. Alloy. Compd..

